# Department of Practice of Medicine

**Published:** 1896-01

**Authors:** 

**Affiliations:** Galveston


### Current Medical Literature.

### DEPARTMENT OF PRACTICE OF MEDICINE.

EDITED BY PROF. ALLEN J. SMITH, M. D., GALVESTON.

DETECTION OF THE DIPHTHERIA BACILLUS BY ITS PECULIAR REACTION TOWARD CERTAIN STAINS.—H. C. Crouch, of Denver (*N. Y. Medical Journal*, Oct. 5, 1895), uses for staining the diphtheritic bacilli either in membrane or in culture, the following solution: Five parts of a fresh one per cent watery solution of methyl green, one part of a fresh watery one per cent solution of dahlia, and four parts of water. If either color predominates add cautiously from the other solution until a true mixed effect is obtained. The mixture improves with age. The cover preparation is prepared in the usaal way, dried and flamed, and a momentary contact with the stain made. After staining for a second—not more—the cover is rinsed in water and may be at once examined. The bacilli will have a faint green tinge, with at each end a well defined round body of a distinctly red color. In the membrane this effect will not be present in all the bacilli; but if shown in even but a few the author's experience justifies him in deciding upon the existence of true diphtheria. In order to bring out the appearance more fully (as it requires a good lense and experienced eye to surely determine the faintly stained bacilli) the author counsels that a subsequent counterstain with a dilute watery solution of Bismarck brown be practiced. Dr. Crouch regards these red bodies in the bacilli as probably of nuclear nature, discrediting the opinions of those who would have them as spores or products of degeneration.

---

GOITRE AMONG THE INDIANS OF THE UNITED STATES.—Dr. E. D. Munson, of the U. S. Army (*N. Y. Med. Journal*, Oct. 26, 1895), had his attention directed to this subject by the great frequency of goitre among the northern Cheyennes; and has since then made a careful study of the matter as presented in the reports of the Bureau of Indian Affairs and from personal experience. He concludes from such study that there is a strong racial predisposition to goitre among Indians; that goitre is a distinctly localized disease; that it does not appear to be caused by high altitude, climate or water containing excess of calcium salts; that it appears to be favored by unsanitary surroundings, depressing constitutional conditions and an improper and excessively nitrogenous diet; that hereditary influence is a prominent factor in its causation: that sex and puberty exert a strong influence in its production, and that there appears to be an intimate relationship in women between the thyroid gland and the reproductive organs; that cretinism is extremely rare in connection with this disease, and that exophthalmic goitre is moderately so; that the tumor is correspondingly smaller than among the whites, and that localities apparently affect the growth of the tumor as well as its frequency.

